# Serum Lipid Profiles and All-Cause Mortality: A Retrospective Single Center Study on Chinese Inpatient Centenarians

**DOI:** 10.3389/fpubh.2022.776814

**Published:** 2022-05-13

**Authors:** Xiao Zou, Jian-hua Li, Yi-xin Hu, Hai-jun Wang, Sha-sha Sun, Wei-hao Xu, Xin-li Deng, Ting Sun, Jian Cao, Li Fan, Quan-jin Si

**Affiliations:** ^1^Cardiology Department of The Second Medical Center and National Clinical Research Center for Geriatric Diseases, Chinese PLA General Hospital, Beijing, China; ^2^The Forth Healthcare Department of The Second Medical Center, Chinese PLA General Hospital, Beijing, China; ^3^Haikou Cadre's Sanitarium of Hainan Military Region, Hainan, China; ^4^Laboratory Department of The Second Medical Center, Chinese PLA General Hospital, Beijing, China; ^5^The Third Healthcare Department of The Second Medical Center, Chinese PLA General Hospital, Beijing, China

**Keywords:** centenarians, lipid-lowering drugs, mortality, risk factor, lipid profiles

## Abstract

**Objectives:**

To analyze the serum lipid profiles and investigate the relationship between the lipoprotein cholesterol levels and all-cause mortality in Chinese inpatient centenarians.

**Design:**

Retrospective study.

**Methods:**

Centenarians aged 100 years and older were admitted from January 2010 to January 2021 in our hospital. All centenarians completed a follow up visit till April 2021 of all-cause mortality and serum lipid profiles, including total cholesterol (TC), triglycerides (TG), low-density lipoprotein cholesterol (LDL-C), high-density lipoprotein cholesterol (HDL-C) levels. Cox proportional hazard models were used to assess the association between lipid profiles and all-cause mortality.

**Results:**

(1) These 121 centenarians on average were 100.85 ± 1.37 years old (100~107 years), including 114 males and 7 females. (2) The rate of treatment with lipid-lowering drugs was 69.4%, and the lipid-lowering drugs were mainly statins (63.6%). (3) The results of serum lipid profiles were as follows: TC 3.90 ± 0.69 mmol/L, TG 1.36 ± 0.55 mmol/L, HDL-C 1.14 ± 0.24 mmol/L, and LDL-C 2.05 ± 0.46 mmol/L. (4) The median follow-up time was 589 days (95% CI: 475, 703), and the all-cause mortality rate was 66.1%. (5) Multivariable analysis showed that higher TC level (HR = 1.968, 95% CI = 1.191–3.253, *P* = 0.008), lower LDL-C level (HR = 0.379, 95% CI = 0.212–0.677, *P* = 0.001) was independent factors contributed to all-cause mortality. Sensitivity analysis showed that the above results were stable. The therapy and complication morbidity did not present significant publication bias.

**Conclusions:**

The serum lipid profiles of Chinese inpatient centenarians were lower than those of the previous studies. Low LDL-C level was associated with an increased risk of all-cause mortality, which may indicate that more intensive lowering of LDL-C had a potential adverse effect on all-cause mortality for centenarians.

## Introduction

The global population is aging, which leads to an increasing burden of age-related cardiology diseases. Centenarians are a specific group with different lipoprotein cholesterol levels compared with those of ordinary elderly people. Atherosclerotic cardiovascular disease (ASCVD) and its clinical manifestations, are the leading causes of morbidity and mortality throughout the world. Lipid levels, especially LDL-C level, have been well established as an important risk factor of ASCVD for decades (1). Evidence has shown that statins could reduce total cardiovascular events, which can improve the prognosis of the elderly patients. What's more, lowering LDL-C level by intensified statin therapy provides incremental additional reduction in cardiovascular risk (2). While studies have shown that (3, 4) the relationship between dyslipidemia and the risk of death will gradually weaken with age, some studies (5–8) have found no association between LDL-C level and the risk of all-cause mortality; lower LDL-C level is not always associated with greater benefit. An excessive low level of LDL-C might be negatively correlated with all-cause mortality (9, 10).

However, contradicting results were reported in the magnitude of the reductions in individual mortality and cardiovascular end points among the elderly patients, especially centenarians. Therefore, the purpose of this study is to analyze the serum lipid profiles and evaluate the relationship between the lipid levels and all-cause mortality in the single center inpatient centenarians.

## Materials and Methods

### Study Population

Centenarians aged 100 years or above and with available clinical data were admitted in our hospital from January 2010 to January 2021, among whom 121 centenarians were qualified for final statistical analysis. The main inclusion criteria into final analysis were (1) the clinical data and laboratory examinations were sufficient; (2) the participants could be successfully followed up successfully till the end point event.

The missing data were excluded for analysis. The key exclusion criteria were as followings: (1) with recent acute coronary syndrome; (2) receiving hemodialysis therapy; (3) Poorly control of blood pressure and glucose; (4) in a serious or critical conditions.

According to “2016 Chinese Guideline for the Management of Dyslipidemia in Adults” (11), the diagnostic criteria for dyslipidemia: 1 Hypercholesteremia: total cholesterol (TC) ≥ 6.20 mmol/L; 2 Hypertriglyceridemia: triglycerides (TG) ≥ 2.3 mmol/L; 3 Combined hyperlipidemia: TC ≥6.20 mmol/L and TG ≥ 2.3 mmol/L; 4 Low level of high-density lipoprotein cholesterol (HDL-C): HDL-C ≤ 1.0 mmol/L; 5 Dyslipidemia: over 1 type of the above conditions.

### Study Design

The clinical data of all admitted participants were exported from Electronic Medical Record, including age, gender, BMI, smoking history, drinking history, comorbid diseases, lipid-lowering drugs, etc. Participants were classified as non-smokers, or current smokers. Non-smokers were defined as adults who had not smoked at least 100 cigarettes in their lifetime, current smokers were defined as adults who had smoked at least 100 cigarettes and were currently smoking. Drinkers were defined as an average alcohol ingestion of ≥30 g/day. BMI was calculated as body weight divided by the square of the height (kg/m^2^). Each chronic disease was defined as described previously. The serum lipid profiles were detected by the immunoturbidimetric method in the biochemical laboratory of our hospital using Roche reagents. TC, TG, LDL-C, HDL-C levels were all recorded till the end of the study.

Methods through outpatient visiting, rehospitalization and telephone, all admitted participants were retrospectively followed up on the day of examination and ended

at the first occurrence of endpoint event, or in April 2021. The time scale for Cox model was 10 years. The endpoint event was defined as all-cause death, referring to the death of any causes that occurred during the follow-up period.

### Statistical Analysis

Continuous variables were presented as mean ± standard deviation, and categorical variables were presented as frequency and percentages.

The final lipid profile levels were calculated according to the average of multiple lipid values during the whole follow-up period. Baseline characteristics between groups were compared by *t*-test or rank sum test for non-normal distribution data, and by Chi-square test for categorical variables.

The survival curves were estimated by the standard Kaplan-Meier estimator, and multivariable Cox models were estimated to investigate associations between serum lipid profiles and all-cause mortality, also used to estimate the adjusted hazard ratios (HRs) and 95% confidence intervals (CIs) of the endpoint events. HR and 95% CIs were obtained by using multivariable Cox models, while beta coefficient (B) and *P*-values were calculated by using linear regression analyses.

The analysis stratified by the lipid-lowering therapy was used to show the association and check whether the findings would be consistent with the whole patients. The Chi-square test was used for the analyses.

The centenarians' data within the recent 5 years were used to evaluate the potential bias, and sensitivity analysis was used to evaluate the stability of the results. The Kaplan-Meier and multivariable Cox models were used for sensitivity analysis.

All statistical analyses were performed with SPSS 22.0 software. A *p*-value of <0.05 was considered as significant difference.

## Results

### The Baseline Features of the Inpatient Centenarians

A total of 121 centenarians, including 114 males and seven females, were eligible for this study. Centenarians on average were 100.85 ± 1.37 years (range: 100–107 years), the majority of centenarians (94.2%) were male. The rate of current smokers and alcoholic drinkers were 1.7 and 9.1% respectively. 84.3% of the patients had coronary heart disease, 80.2% with hypertension, 31.4% with cancer, and 40.5% with diabetes mellitus. There were 37 cases (30.6%) diagnosed as dyslipidemia, and 70.5% of the patients using lipid-lowering drugs (seen in Table 1).

**Table 1:** The baseline features of inpatient centenarians.

**Items**	**Total (*n* = 121)**	**Dyslipidemia group (*n* = 37)**	**Normal group (*n* = 84)**	***P-value***
Age (years)	100.85 ± 1.37	100.92 ± 1.44	100.82 ± 1.35	0.72
BMI (kg/m^2^)	22.72 ± 3.18	22.84 ± 3.46	22.66 ± 3.07	0.79
Gender (M/F)	114/7	37/0	77/7	0.099
Smokers [*n* (%)]	2 (1.7)	2 (5.4)	0 (0)	0.092
Drinkers [*n* (%)]	11 (9.1)	5 (13.5)	6 (7.1)	0.308
CHD [*n* (%)]	102 (84.3)	30 (81.1)	72 (85.7)	0.519
Hypertension [*n* (%)]	97 (80.2)	29 (78.4)	68 (81.0)	0.744
DM [*n* (%)]	49 (40.5)	16 (43.2)	33 (39.3)	0.683
Cancer [*n* (%)]	38 (31.4)	9 (24.3)	29 (34.5)	0.265
Lipid-lowering	84 (69.4)	29 (78.4)	55 (65.5)	0.156
Drugs [*n* (%)]				
Statins [*n* (%)]	77 (63.6)	24 (64.9)	53 (63.1)	0.852
Fibrates [*n* (%)]	3 (2.5)	2 (5.4)	1 (1.2)	0.221
Niacin [*n* (%)]	1 (0.8)	1 (2.7)	0 (0)	0.306
Ezetimibe [*n* (%)]	2 (1.7)	1 (2.7)	1 (1.2)	0.52
Policosanol [*n* (%)]	3 (2.5)	2 (5.4)	1 (1.2)	0.221

*BMI, body mass index; CHD, coronary heart disease; DM, diabetes mellitus. P-value is for comparison between dyslipidemia group and normal group*.

### The Serum Lipid Levels of Inpatient Centenarians

The serum lipid levels of these 121 centenarians were as follows: TC 3.90 ± 0.69 mmol/L, TG 1.36 ± 0.55 mmol/L, HDL-C 1.14 ± 0.24 mmol/L, LDL-C 2.05 ± 0.46 mmol/L.

Centenarians were divided into two groups by the difference of having dyslipidemia or not. Compared with normal group, the TG level in the dyslipidemia group was significantly higher, while the HDL-C level was significantly lower (both *P* < 0.05). There were no significant differences in TC and LDL-C levels between the two groups (Table 2).

**Table 2:** The serum lipid levels between the two groups.

**Items**	**Total**	**Dyslipidemia group**	**Normal group**	***P-value***
	**(*n* = 121)**	**(*n* = 37)**	**(*n* = 84)**	
TC (mmol/L)	3.90 ± 0.69	3.74 ± 0.71	3.97 ± 0.68	0.09
TG (mmol/L)	1.36 ± 0.55	1.70 ± 0.62	1.22 ± 0.44	0.00
HDL-C (mmol/L)	1.14 ± 0.24	0.88 ± 0.14	1.26 ± 0.18	0.00
LDL-C (mmol/L)	2.05 ± 0.46	2.05 ± 0.48	2.04 ± 0.46	0.89

*TC, total cholesterol; TG, triglyceride; HDL-C, high-density lipoprotein cholesterol; LDL-C, low-density lipoprotein cholesterol. P-value is for comparison between dyslipidemia group and normal group*.

### Associations of LDL-C Level With All-Cause Mortality

**Figure 1:**
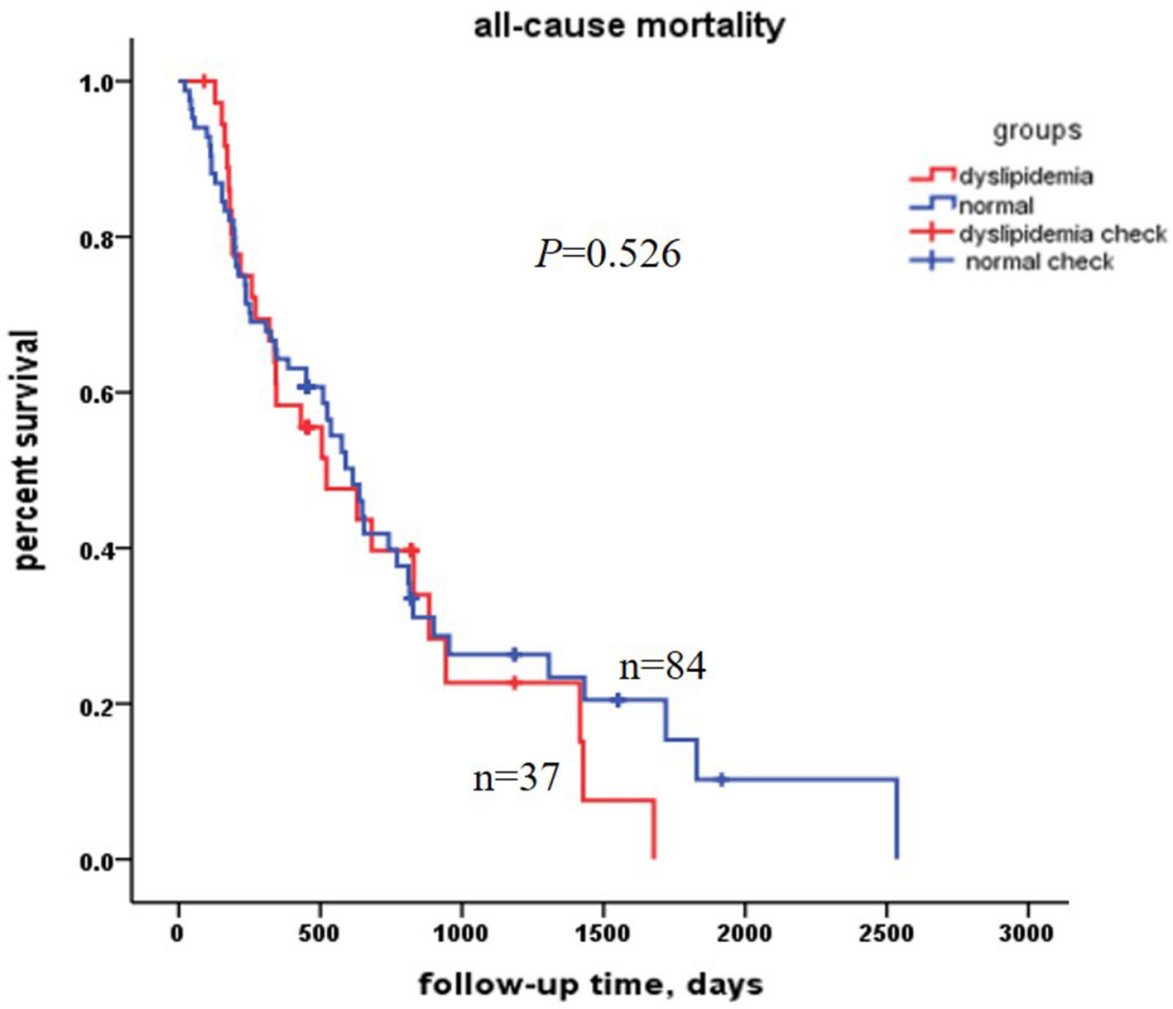
Kaplan–Meier survival curves estimation for all-cause mortality in the 121 centenarians. There was no significant difference between the dyslipidemia centenarians and the normal centenarians.

The follow-up duration ranged from 21 days to 2,535 days, and the median follow-up time was 589 days (95% CI: 475, 703). A total of 80 out of 121 centenarians died during this period, leading to an all-cause mortality rate of 66.1%. So far, 41 cases (33.9%) were still alive. The median follow-up was 521 days (95% CI: 234, 808), and 615 days (95% CI: 494,736) for the dyslipidemia and the normal group, respectively. The Kaplan–Meier survival curves for all-cause mortality in the dyslipidemia group and the normal group were shown in Figure 1, indicating no significant difference of the survival probability in the two groups (*P* = 0.526).

**Figure 2:**
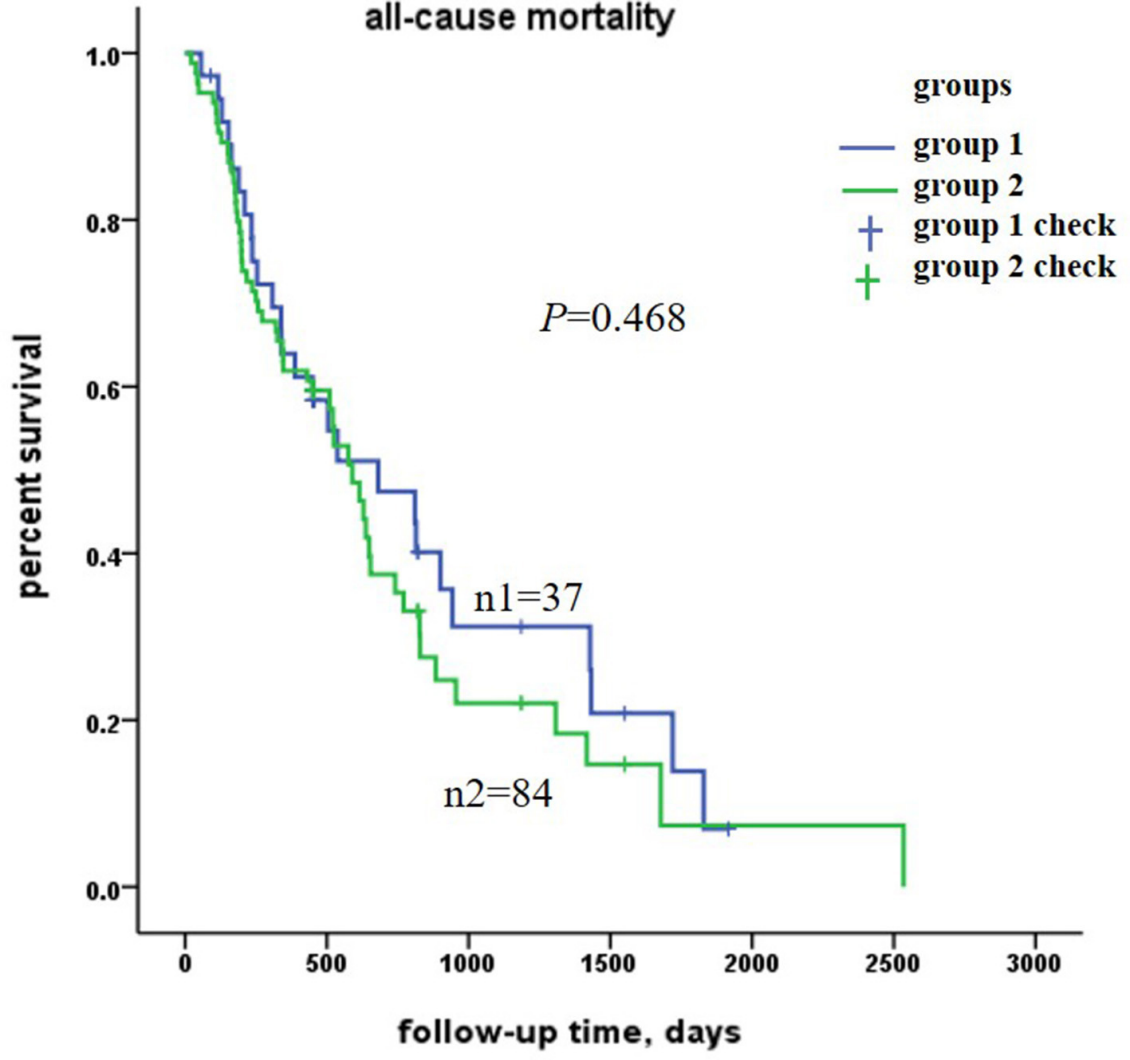
Kaplan–Meier survival curves estimation for all-cause mortality in the 121 centenarians. There was no significant difference between group 1 and group 2. Group 1 was for lipid lowering drugs non-users, Group 2 was for lipid lowering drugs users.

The analysis stratified by the lipid-lowering therapy was used to show the association and check whether the findings would be consistent between the two groups. The Chi-square test showed there was no significant difference of all-cause mortality between the lipid lowering drugs non-users and lipid lowering drugs users (*P* = 0.468), and the Kaplan–Meier survival curves showed the same result (Figure 2).

Multivariable analysis was adjusted by age, BMI, coronary heart disease, diabetes mellitus, hypertension, cancer and the lipid-lowering therapy. We found that TC level in centenarians was a risk factor (HR = 1.968, 95% CI = 1.191–3.253, *P* = 0.008) for all-cause death, while LDL-C level (HR = 0.379, 95% CI = 0.212–0.677, *P* = 0.001) was the protective factor for all-cause death (see Table 3). It suggested that low LDL-C level might be associated with an increased risk of all-cause in centenarians.

**Table 3:** The multivariate analysis in 121 centenarians.

**Items**	**B**	**SE**	**Wald**	**Sig**.	**HR**	**95.0% CI**	
						**lower**	**upper**
Age	−1.768	1.064	2.761	0.097	0.171	0.021	1.374
BMI	−0.039	0.040	0.950	0.330	0.962	0.890	1.040
Smoking	0.910	1.056	0.741	0.389	2.483	0.313	19.685
Drinking	−0.324	0.415	0.610	0.435	0.723	0.321	1.631
TC	0.677	0.256	6.983	0.008	1.968	1.191	3.253
TG	−0.033	0.309	0.011	0.916	0.968	0.528	1.772
HDL-C	−1.267	0.749	2.860	0.091	0.282	0.065	1.223
LDL-C	−0.971	0.296	10.756	0.001	0.379	0.212	0.677
Cancer	0.519	0.260	3.992	0.046	1.680	1.010	2.796
CHD	0.201	0.370	0.296	0.587	1.223	0.592	2.527
Hypertension	0.414	0.350	1.398	0.237	1.513	0.762	3.006
DM	0.302	0.249	1.478	0.224	1.353	0.831	2.203
Lipid-lowering therapy	0.080	0.283	0.079	0.778	1.083	0.622	1.886

*BMI, body mass index; TC, total cholesterol; TG, triglyceride; HDL-C„ high-density lipoprotein cholesterol; LDL-C, low-density lipoprotein cholesterol; CHD, coronary heart disease; DM, diabetes mellitus*.

### Results of the Sensitivity Analysis

**Figure 3:**
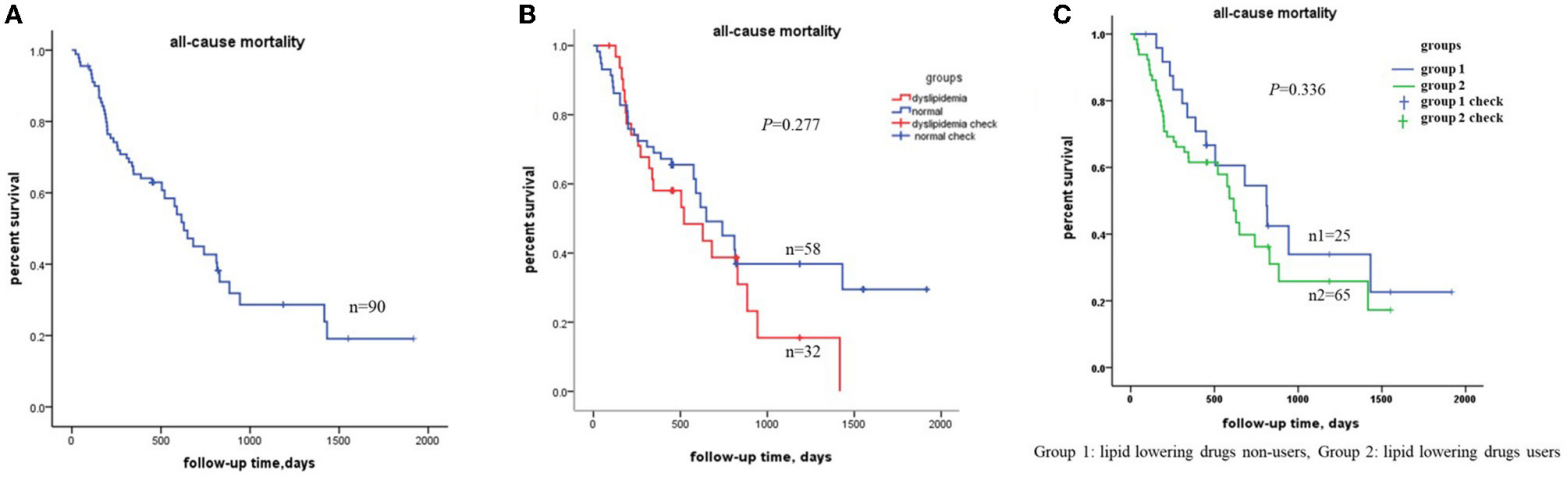
Kaplan–Meier survival curves estimation in the 90 centenarians from recent 5 years (from 2016 to 2021). **(A)** The total all-cause mortality in the 90 centenarians; **(B)** the total all-cause mortality between the dyslipidemia group and normal group; **(C)** the all-cause mortality stratified by the lipid-lowering therapy. Group 1 was for lipid lowering drugs non-users, Group 2 was for lipid lowering drugs users. Both *P* > 0.05, which indicated the same results as the whole centenarians.

There were 90 out of 121 centenarians from the recent 5 years, from2016 to 2021. The 90 centenarians were also divided into two groups: dyslipidemia group and normal group. There were 49 out of 90 centenarians died during this period, leading to an all-cause mortality rate of 54.4%. The Kaplan–Meier survival curves were showed in Figure 3. The multivariable analysis showed that TC level was a risk factor and LDL-C level was the protective factor for all-cause death, which were the same as the whole 121 centenarians (see Table 4). The analyses stratified by the lipid-lowering therapy were undertaken to check the findings, and the results were consistent with the two groups. Sensitivity analysis showed that the above results were stable.

**Table 4:** The multivariable analysis in the 90 centenarians from the recent 5 years.

**Items**	**B**	**SE**	**Wald**	**Sig**.	**HR**	**95.0% CI**	
						**lower**	**upper**
TC	0.754	0.377	4.008	0.045	2.126	1.016	4.451
TG	0.421	0.388	1.180	0.277	1.524	0.713	3.258
HDL-C	−0.478	0.875	0.299	0.585	0.620	0.112	3.444
LDL-C	−1.615	0.464	12.137	0.000	0.199	0.080	0.493

*TC, total cholesterol; TG, triglyceride; HDL-C, high-density lipoprotein cholesterol; LDL-C, low-density lipoprotein cholesterol. The analysis was adjusted by age, BMI, coronary heart disease, diabetes mellitus, hypertension, cancer, and the lipid-lowering therapy*.

## Discussion

In this study, the average levels of TC, TG, LDL-C and HDL-C in inpatient centenarians were 3.90 ± 0.69 mmol/L, 1.36 ± 0.55 mmol/L, 2.05 ± 0.46 mmol/L and 1.14 ± 0.24 mmol/L, respectively. Previous studies have shown that the incidence of dyslipidemia in Chinese adults was 26.3–42.65% (12, 13). The latest research on distribution characteristics of blood lipid profile in Hainan centenarians (14) showed that the median levels of TC, TG, LDL-C and HDL-C were 4.60 mmol/L, 1.05 mmol/L, 2.77 mmol/L and 1.41 mmol/L, respectively, and the prevalence of dyslipidemia was 19.1%. Centenarians in Hainan were all community populations, while centenarians in our study were all from hospital. The levels of TC and LDL-C in this study were lower comparing to the previous studies, which may be related to multiple reasons, such as the coexistence of chronic diseases in hospitalized patients, combination effect of lipid-lowering drugs and other non-drug therapies.

In this study, 69.4% of centenarians had taken lipid-lowering drugs in hospital, and the lipid-lowering drugs were mainly statins (63.6%). Statins are known to reduce LDL-C level by roughly 50% and, decrease cardiovascular risks, which were recommended for primary and secondary drugs for preventions of ASCVD (11). Previous studies have shown that the treatment rate of lipid-lowering drugs in China was only 5~14.5% (12). However, the use of statins in patients with acute coronary syndrome in China have increased greatly in recent years (15). The treatment rate of lipid-lowering drugs was higher than previous studies mainly related to the better compliance of drugs in hospital, which indicated that the treatment rate of lipid-lowering drugs for the elderly can be improved by strengthening publicity, education and supervision.

We investigated the relationship between LDL-C level and 10 years all-cause mortality among inpatient centenarians in this study. We found that LDL-C level was a protective factor for all-cause death (HR = 0.379, 95% CI = 0.212–0.677, *P* = 0.001). And the sensitivity analysis showed that our results were stable. Previous studies have generally focused on that more intensive LDL-C lowering can reduce the risk of all-cause and cardiovascular mortality for ASCVD patients (16). The lipid status and the association between LDL-C level and all-cause mortality in inpatient centenarians are still poorly evaluated. Studies have confirmed that lowering of LDL-C with standard statin regimens can reduce the risk of occlusive vascular events in ASCVD patients. More intensive lowering of LDL-C level with statin therapy also produced definite further reductions in the incidence of cardiac events (8, 17). A meta-analysis of 34 studies (16) showed that intensive lipid-lowering therapy is beneficial to the end points. In this meta-analysis, compared with less intensive LDL-C lowering, more intensive reduction was associated with a greater benefit to cardiovascular mortality in patients with higher baseline LDL-C levels. But this association was not found when baseline LDL-C level was < 100 mg/dl. The direct association of LDL-C level with all-cause mortality was still unclear. A nationwide representative longitudinal study based on the data from the China Health and Retirement Longitudinal Study (CHARLS), found some different results from previous studies (18); this study demonstrated that middle-aged and elderly Chinese men with very low LDL-C level had an increased risk of all-cause mortality. Similarly, a Chinese recent study (19) of 6,941 elderly people showed that the ratio of LDL-C/HDL-C level had a U-shaped relationship with all-cause mortality in hypertensive patients over 65-year-old, suggesting that both lower and higher LDL-C/HDL-C ratios will all increase the all-cause mortality. In addition, previous study showed the LDL-C level of 3.6 mmol/L (140 mg/ dl) may have the lowest risk of all-cause mortality (20). Recently, a study form Korean (21) in the elderly population found LDL-C level was not correlated with cardiovascular death and all-cause death, indicating high LDL-C level may not be a risk factor for CVD in the elderly individuals.

## Conclusion

The results in our study proved the point that “lowest was not the best”, suggesting the potential harmful effect of more intensive lowering of LDL-C on all-cause mortality, which indicates that special attention should be paid to the inpatient centenarians on proper LDL-C level. However, prospective and well-designed cohort studies are still needed to validate the relationship between LDL-C level and mortality in centenarians.

## Study Limitations

There were some limitations in this study. (1) The association between LDL-C level and mortality of subgroups could not be analyzed due to the small sample size of centenarians in a single center; centenarians from multiple-center should be continuously admitted to expand the sample size to verify the reliability of the results. (2) The centenarians were often complicated by physical comorbidity and polypharmacy, and we were failed to collect all the drugs details so that we can't analyze the confounding factors. However, the study is still on-going, and further analysis should be carried out when the sample size is qualified. Then we will check the correlation between combination of different drugs and all-cause mortality.

## Data Availability Statement

The raw data supporting the conclusions of this article will be made available by the authors, without undue reservation.

## Ethics Statement

The studies involving human participants were reviewed and approved by Chinese PLA General Hospital Ethics Committee. The patients/participants provided their written informed consent to participate in this study.

## Author Contributions

XZ, LF, and QJS: conceptualization. XZ, YXH, and JHL: patients enrolling. XZ, JHL, HJW, SSS, TS, and XLD: data collecting and follow-up. XZ and WHX: statistical analysis. XZ: writing—original draft and funding acquisition. XZ, QJS, LF, and JC: writing—review and editing. All authors contributed to the article and approved the submitted version.

## Funding

This work was supported by the Second Medical Center Foundation of Chinese PLA General Hospital (ZXFH2003).

## Conflict of Interest

The authors declare that the research was conducted in the absence of any commercial or financial relationships that could be construed as a potential conflict of interest.

## Publisher's Note

All claims expressed in this article are solely those of the authors and do not necessarily represent those of their affiliated organizations, or those of the publisher, the editors and the reviewers. Any product that may be evaluated in this article, or claim that may be made by its manufacturer, is not guaranteed or endorsed by the publisher.
